# Debulking surgery for functional pleural dissemination of parathyroid carcinoma-case report

**DOI:** 10.1186/s13019-021-01477-z

**Published:** 2021-04-15

**Authors:** Sachi Kawagishi, Soichiro Funaki, Naoko Ose, Kenji Kimura, Kosuke Mukai, Michio Otsuki, Yasushi Shintani

**Affiliations:** 1 Department of General Thoracic Surgery, Osaka University Graduate School of Medicine, 2-2-L5, Yamadaoka, Suita, 565-0871 Osaka, Japan; 2 Department of Metabolic Medicine, Osaka University Graduate School of Medicine, Suita, Osaka, Japan

**Keywords:** Parathyroid carcinoma, Primary hyperparathyroidism, Pleural metastasis, Debulking surgery

## Abstract

**Background:**

A rare cause of primary hyperparathyroidism (PHPT) is a parathyroid carcinoma. Hypercalcemia with an elevated parathyroid hormone (PTH) level seen in recurrent and metastasis disease cases is often refractory to medical therapy, thus surgical resection is recommended when possible. We performed debulking surgery for pleural dissemination of parathyroid cancer for improvement of symptoms in a patient with hypercalcemia.

**Case presentation:**

A 30-year-old male with hypercalcemia was diagnosed with parathyroid cancer. Following surgery, intact PTH level elevation and hypercalcemia progression due to recurrent disease were noted. An active status of functional left pleural dissemination was revealed in 99mTc-methoxyisobutylisonitrile and somatostatin receptor scintigraphy results, but not in the area of pulmonary metastasis, and debulking surgery was performed. Thereafter, the PTH level was decreased temporarily and activities of daily living improved.

**Conclusion:**

Aggressive resection of metastatic disease in patients with a parathyroid carcinoma is taken into consideration to control hypercalcemia.

## Background

A parathyroid carcinoma is a rare cause of primary hyperparathyroidism (PHPT), accounting for less than 1% of all reported cases. Treatment for parathyroid cancer is generally surgical resection, though previous reports indicate that affected patients often develop recurrence and metastasis after surgery, and show a poor prognosis because of hypercalcemia and subsequent metabolic complications rather than tumor invasiveness [1]. Hypercalcemia with an elevated parathyroid hormone (PTH) level seen in recurrent and metastasis disease cases is often refractory to medical therapy, thus surgical resection is recommended when possible [2]. Herein, we report details of a patient who underwent a debulking procedure for recurrence of pleural dissemination following surgery for a parathyroid carcinoma.

## Case presentation

A 30-year-old male visited another hospital with dyspnea, polydipsia, and polyuria, as well as vomiting and joint pain, and had experienced weight loss of 25 kg during the previous year. Laboratory testing showed serum calcium at 20.6 mg/dL and PTH elevated to 10,380 pg/mL, and parathyroid cancer was strongly suspected after imaging was performed. The patient was referred to our hospital, where en bloc resection of the tumor located along the left lobe of the thyroid gland and neck dissection were performed, and pathological findings confirmed a diagnosis of parathyroid cancer. Following surgery, radiation therapy with 66 Gy was performed. Thereafter, PTH was 100 pg/mL and the patient was followed in an outpatient setting.

**Fig. 1:**
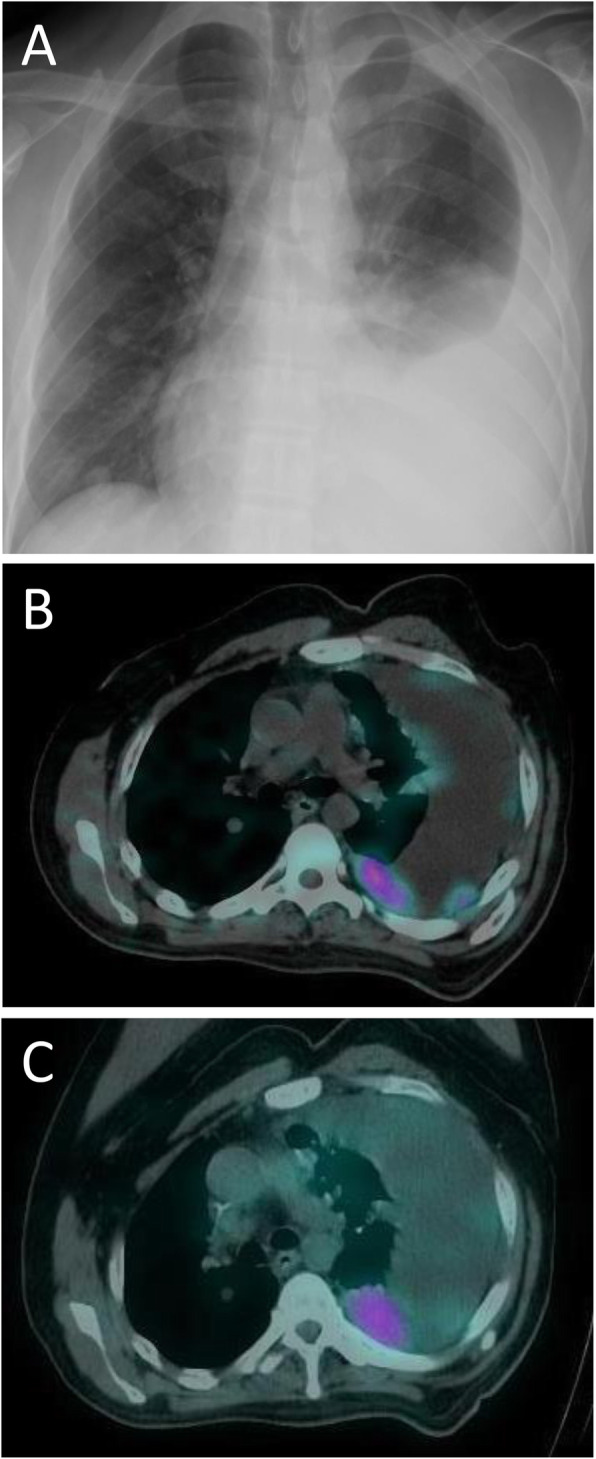
Preoperative imaging findings. **a** Chest X-ray showing massive pleural effusion. **b** MIBI scintigraphy and **c** somatostatin receptor scintigraphy showing functionally active status in left pleural dissemination, but not in the area of pulmonary metastasis

At 32 months after surgery, blood test results showed re-elevation of PTH, and chest computerized tomography (CT) revealed bilateral multiple lung nodules and left pleural dissemination. Symptoms of hypercalcemia also progressed, thus the patient required hospitalization because of progression of subjective symptoms. Treatments for hypercalcemia with intravenous hydration, diuretics, and administration of cinacalcet (a second-generation calcimimetic), as well as denosumab (fully human monoclonal antibody that binds the cytokine receptor activator of NFκB ligand), octreotide (long-acting somatostatin analogue), and zoledronic acid (bisphosphonate) were performed. However, elevation of PTH level and hypercalcemia progressed. Chest X-ray findings showed massive pleural effusion (Fig. 1a). Results obtained with both 99mTc-methoxyisobutylisonitrile (MIBI) scintigraphy and somatostatin receptor scintigraphy revealed functionally active status lesions in left pleural dissemination, but not in the area of pulmonary metastasis (Fig. 1b, c). Radiation therapy with 60 Gy for left pleural dissemination was performed, though response was poor. The patient was then referred to our department for surgical resection of left pleural dissemination at 37 months after the initial surgery.

**Fig. 2:**
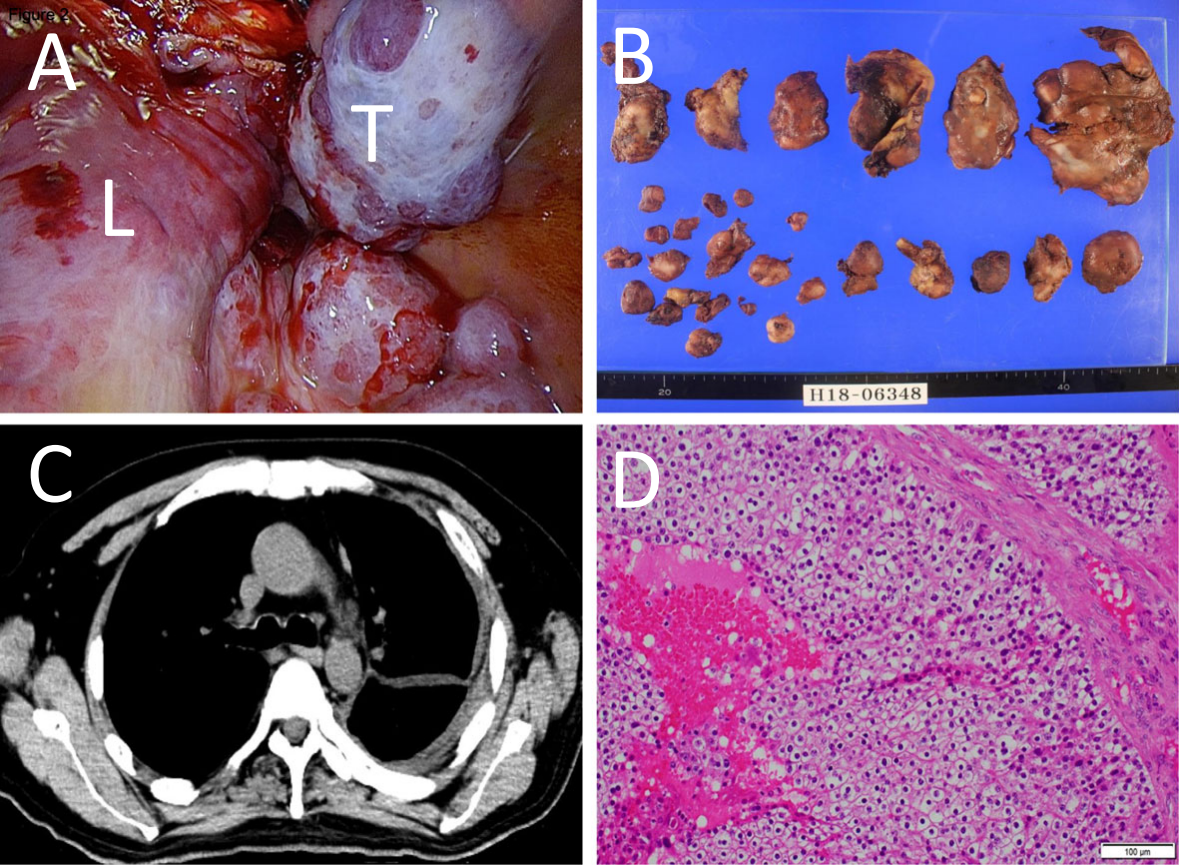
**a** Intraoperative findings. Several tumors 3–5 cm in size were found in the left thoracic cavity and resected as much as possible via a thoracotomy. **b** Images of resected tumor. **c** Following resection. Chest CT showed that the functionally active site of left pleural dissemination was nearly completely excised. T, tumor; L, lung. **d** Pleural dissemination shown by hematoxylin and eosin staining. Tumor cells with round nuclei and lightly acidophilic cytoplasm showed a solid pattern (× 200)

Volume reduction of left pleural dissemination to control hypercalcemia was performed via 5th intercostal space thoracotomy. There were several hypervascular tumors 3–5 cm in size disseminated throughout the left thoracic cavity. The pleural tumor was resected using parietal extra-pleural dissection as much as possible. (Fig. 2a, b). We cut into the tumors in order not to damage the pulmonary parenchyma. We cut into the tumors so as to not damage the pulmonary parenchyma. The edges of the tumors on the parietal and visceral pleura were cauterized for hemostasis, while ablation was also done using a soft coagulation system that delivers a computer-controlled low voltage level without electrical discharge. The operation duration was 240 min and blood loss was 3100 mL. The pleural tumors were hypervascular. Also, we could not observe tumors in the pleural cavity at first, as they were disseminated throughout the left thoracic cavity, thus parietal extrapleural dissection was performed to remove the tumor causing massive bleeding. Chest CT results after resection showed that the functionally active site of left pleural dissemination was nearly completely excised (Fig. 2c). A postoperative pathological examination revealed findings similar to parathyroid cancer, thus the diagnosis was pleural dissemination of parathyroid cancer (Fig. 2d). PTH level was 46,800 pg/mL prior to resection of pleural dissemination and then decreased to 7636 pg/mL on the first postoperative day. Infusion volume was gradually decreased and the serum calcium level did not increase. Hypercalcemia symptoms were improved and the patient was discharged 56 days later. Under medical treatment for hypercalcemia with denosumab and cinacalcet, the PTH level gradually increased, while serum calcium level did not change. Eight months after the resection of pleural dissemination, the patient died at home from cachexia related to the disease.

## Discussion and conclusions

The most common cause of PHPT is parathyroid adenoma, accounting for 90% of all cases. On the other hand, a parathyroid carcinoma is a rare type of endocrine malignancy, accounting for < 1% of all cases of PHPT, most of which show general symptoms of hypercalcemia as seen in the present patient [3]. A blood test at the time of diagnosis reveals elevated serum calcium and PTH levels to a significantly greater level in patients with parathyroid cancer as compared to those with parathyroid adenoma [4]. Some reports have noted that serum PTH serves as an excellent indicator of recurrence and metastasis after initial surgery for parathyroid cancer [5]. MIBI scintigraphy reveals uptake of PHPT, thus is useful for localization of ectopic diseases and distant matastasis [1]. Also, since a parathyroid carcinoma expresses somatostatin receptors, scintigraphy may be beneficial for showing that expression and distant metastasis related to the tumor [6].

Chemotherapy has not been demonstrated to be beneficial for parathyroid carcinoma treatment [3]. Furthermore, a parathyroid carcinoma and sites of distant metastasis are generally not considered to be radiosensitive, thus complete resection of an isolated metastatic site or pulmonary metastases after initial surgery for parathyroid cancer can control serum calcium and PTH levels [5].

To the best of our knowledge, this is the first report of debulking surgery for a patient with a wide range of pleural dissemination of parathyroid cancer. The point is that the degree of MIBI or somatostatin receptor scintigraphy uptake was useful to expect PTH production capacity of these metastatic regions in this patient. We considered that the PTH-producing site was mainly pleural dissemination and performed resection of that as much as possible. Therefore, PTH level revealed 84% reduction after debulking surgery and we were able to decrease serum calcium level. Although debulking surgery for pleural dissemination did not achieve complete remission, discharge from the hospital to receive home medical care was possible because of improvements in subjective symptoms. Radiation therapy had poor effects in this case. It was considered that the subjective symptoms would not improve and patient prognosis would be very poor if only internal treatments such as chest drainage and pleurodesis were performed.

In conclusion, aggressive resection of metastatic disease in patients with a parathyroid carcinoma is taken into consideration to control hypercalcemia. MIBI scintigraphy and somatostatin receptor scintigraphy may be beneficial for identification of a PTH-producing site in such cases.

## Data Availability

Not applicable.

## Abbreviations

CT: Computerized tomography
MIBI: Methoxyisobutylisonitrile
PHPT: Primary hyperparathyroidism
PTH: Parathyroid hormone
